# Fabry–Perot Cavity Sensing Probe with High Thermal Stability for an Acoustic Sensor by Structure Compensation

**DOI:** 10.3390/s18103393

**Published:** 2018-10-10

**Authors:** Jin Cheng, Yu Zhou, Xiaoping Zou

**Affiliations:** 1 Research Center for Sensor Technology, Beijing Key Laboratory for Sensor, Ministry of Education Key Laboratory for Modern Measurement and Control Technology, School of Applied Sciences, Beijing Information Science and Technology University, Beijing 100101, China; xpzou2014@163.com; 2 College of Underwater Acoustic Engineering, Harbin Engineering University, Harbin 150001, China; zycetc3@163.com; 3 Third Research Institute of China Electronics Technology Group Corporation, Beijing 100015, China

**Keywords:** Fabry–Perot cavity, thermal expansion model, structure compensation, acoustic sensor, high thermal stability

## Abstract

Fiber Fabry–Perot cavity sensing probes with high thermal stability for dynamic signal detection which are based on a new method of structure compensation by a proposed thermal expansion model, are presented here. The model reveals that the change of static cavity length with temperature only depends on the thermal expansion coefficient of the materials and the structure parameters. So, fiber Fabry–Perot cavity sensing probes with inherent temperature insensitivity can be obtained by structure compensation. To verify the method, detailed experiments were carried out. The experimental results reveal that the static cavity length of the fiber Fabry–Perot cavity sensing probe with structure compensation hardly changes in the temperature range of −20 to 60 °C and that the method is highly reproducible. Such a method provides a simple approach that allows the as-fabricated fiber Fabry–Perot cavity acoustic sensor to be used for practical applications, exhibiting the great advantages of its simple architecture and high reliability.

## 1. Introduction

The fiber Fabry–Perot cavity (FFPC) is an outstanding structure for sensing applications. Sensors based on FFPC are highly sensitive, resistant to electromagnetic interference, and can work in hostile environments, which are just some of the advantages of all fiber sensors. Besides, FFPC sensors have some unique characteristics, such as being simple, responsive, precise, versatile, and immune to environmental noise [1]. Among fiber sensors, they appear to be very promising, and various FFPC sensors have been developed in recent years, such as magnetic field sensors [2,3], humidity and temperature sensors [4,5,6], acoustic wave sensors [7,8], refractive index sensors [9,10], pressure sensors [11,12], strain sensors [13,14], vibration sensors [15,16], liquid level sensors [17,18], etc.

For dynamic signal detection, such as for sound waves, where the light source is monochromatic, the FFPC sensor should work at a quadrature point of the interference spectrum [19,20,21,22,23] to achieve a linear output and high sensitivity. This method is called intensity demodulation [22], which requires the FFPC sensing probe to have a certain cavity length. 

However, the cavity length varies with changes of the surrounding circumstances, especially as the temperature changes [24]. It is a fatal problem when the working point drifts away from the quadrature, which leads to a decrease in the sensitivity of the FFPC sensor. At present, three main methods have been developed to resolve this critical problem. For the first method, sensors are made of materials with a low thermal expansion coefficient. For example, Wang et al. tested an all-silica FFPC pressure sensor. The temperature dependence of the pressure sensor was measured to be as low as 0.009%/°C of full reading [21]. Second, sensors have been proposed that have a feedback circuit to compensate the change of the cavity length by tuning the working wavelength of the light. For example, Chen et al. proposed a stabilization method with active feedback control of the diode laser to prevent drifting of the quadrature point [25]. Wang et al. proposed a feedback stabilization technique to control the output wavelength of the tunable fiber laser to operate in the linear range [26]. Mao et al. described a stabilizing operation point technique based on a tunable distributed feedback laser for quadrature demodulation of interferometric sensors [27]. Third, the output signal of sensors is demodulated by an orthogonal signal demodulation algorithm. The key to this method is to obtain an orthogonal signal. For instance, Kim et al. proposed a dual-cavity fiber Fabry–Perot interferometer with a phase-compensating algorithm, the initial phase difference of which between two sinusoidal signals was induced from an interferometer that was automatically adjusted to exactly 90 degrees [28]. Vetrov et al. reported a method for signal reconstruction that used two channels with different wavelengths with the phase shift of the signals in these channels being π/2 [29]. Apparently, most of these proposals have some drawbacks, such as the high cost of the tunable laser and dual-wavelength source, a complicated system of dual-cavity structure and orthogonal signal demodulation, and insensitivity to low-frequency sound waves for the feedback system [25].

In this paper, we present a new simple but effective structure compensation method to get a temperature-insensitive FFPC sensing probe based on a thermal expansion model of FFPC probes. This method gives the probe inherent thermal stability. Therefore, the optical fiber sensor based on the method has the advantages of having a simple system, low cost, and the ability to respond to low-frequency signals. According to our proposed thermal expansion model, the key to structure compensation mainly involves the control of the thermal expansion coefficient of the materials and adjusting the static cavity length of FFPC. By applying structure compensation to an FFPC acoustic sensing probe, FFPC acoustic sensors with high thermal stability were obtained in the temperature range of −20 to 60 °C. This meets the requirements for practical applications.

## 2. Thermal Expansion Model and Structure Design

**Figure 1:**
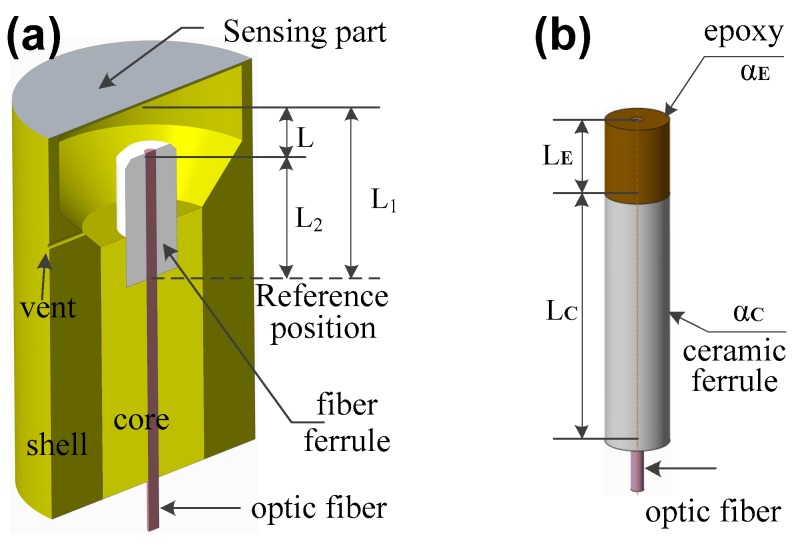
Structure of the fiber Fabry–Perot cavity (FFPC) sensing probe (**a**), composite ferrule of epoxy, and ceramic fiber ferrule (**b**).

The structure of the FFPC sensing probe is shown in Figure 1a. The FFPC has two reflectors that reflect light back to the fiber to form interference: one is the top surface of the optic fiber, the other is the inner surface of the sensing part. The sensing part could be a membrane, mass, or other sensing materials that can detect sound waves, vibrations, and acceleration. Due to the low reflectivity of the fiber tip surface, FFPC interference can be simplified as two-beam light interference. Therefore, the intensity of the interference light can be expressed by Equations (1)–(3) [30]:

*I* = *I*_0_[1 + *γ*cos(*φ*_0_ + Δ*φ*)] (1)

*φ*_0_ = 4π*nL*/*λ* (2)

Δ*φ* = *φ*_m_cos(*ωt* − *ψ*_0_) (3)

where *I*_0_ = (*I*_max_ + *I*_min_)/2, *I*_max_ and *I*_min_ denote the maximum and minimum power of the interference light, *γ* = (*I*_max_ − *I*_min_)/(*I*_max_ + *I*_min_) is the fringe contrast, *φ*_0_ is the static phase difference between the two reflected lights, L is the static cavity length of FFPC, *λ* is the wavelength of the laser, *n* is the refractive index of the medium (here, this is air, *n* ≈ 1), Δ*φ* is the phase change caused by a dynamic signal, *φ*_m_ is the amplitude of Δ*φ*, and ω and *ψ*_0_ are the round frequency and initial phase of Δ*φ*. 

For low-fineness Fabry–Perot interference, when the change of the cavity length reaches *λ*/8, the working point has a drift of π/2. This can lead to a dramatic decrease in sensitivity. For *λ* = 1552 nm, *λ*/8 is only 194 nm. The change of the cavity length easily reaches this value with the temperature change. For example, when the sensor is made of a nickel–copper alloy, the length change is about 144 nm per millimeter at the temperature change of 10 °C. This means that the cavity length is easily affected by temperature, as mentioned above.

To understand the effect of temperature on the static cavity length, the relationship between the static cavity length and temperature must be derived. The main components of the probe include the shell, core, sensing part, fiber ferrule, and optic fiber, as shown in Figure 1a. The thermal expansion coefficient of the fiber is about 0.55 × 10^−6^ m/(m·°C). This is far less than that of the ferrule and the shell. The thermal effect of the fiber can be neglected. The materials of the shell and core were selected to be the same, and their thermal expansion coefficients are referred as *α*_1_. The thermal expansion coefficient of the fiber ferrule is referred as *α*_2_ and the bottom of the fiber ferrule is referred to as a reference position for all length.

From Figure 1a, the static cavity length L can be expressed as

*L* = *L*_1_ − *L*_2_ (4)

where *L*_1_ is the length from the top surface of the shell to the reference position, and *L*_2_ is the length from the top surface of the fiber ferrule to the reference position.

It is considered that the static cavity length L is a function of *α*_1_, *α*_2_, *L*_1_, *L*_2_, and temperature *T*, and thus, the change rate of the static cavity length with temperature can be described as follows:

(5) $$\frac{dL}{dT}=\frac{\partial L}{\partial \alpha_{1}}\frac{\partial \alpha_{1}}{\partial T}+\frac{\partial L}{\partial \alpha_{2}}\frac{\partial \alpha_{2}}{\partial T}+\frac{\partial L}{\partial L_{1}}\frac{\partial L_{1}}{\partial T}+\frac{\partial L}{\partial L_{2}}\frac{\partial L_{2}}{\partial T}\;$$

Assuming that all thermal expansion coefficients are constant over certain temperature ranges, then

(6) $$\frac{\partial \alpha_{1}}{\partial T}=0,\;\frac{\partial \alpha_{2}}{\partial T}=0\;$$

According to the theory of thermal expansion, there is

(7) $$\frac{\partial L_{1}}{\partial T}=\alpha_{1}L_{1},\;\frac{\partial L_{2}}{\partial T}=\alpha_{2}L_{2}\;$$

From Equation (4), the following values can be obtained:

(8) $$\frac{\partial L}{\partial L_{1}}=1,\;\frac{\partial L}{\partial L_{2}}=1\;$$

Then, *dL*/*dT* can be derived from Equations (5)–(8) as

(9) $$\frac{dL}{dT}=\alpha_{1}L_{1}-\alpha_{2}L_{2}\;$$

Equation (9) is a thermal expansion model of the FFPC probe. It reveals that the change of static cavity length with temperature only depends on the thermal expansion coefficient of the materials and the structure parameters. 

Obviously, the value of *dL*/*dT* could be more than zero, less than zero, or equal to zero. In the case of *dL*/*dT* > 0 or *dL*/*dT* < 0, the static cavity length increases or decreases with increasing temperature, respectively. These situations are not expected to happen; however, it is almost a fact [16,17,18,19]. For *dL*/*dT* = 0, the static cavity length does not change with temperature change, which indicates that the probe has an intrinsically high thermal stability, which is what we wish to achieve. 

It can be seen from Equation (9), in the case of *α*_1_ ≥ *α*_2_, *dL*/*dT* is always greater than zero because of *L*_1_ ≥ *L*_2_. If it is desired to get *dL*/*dT* = 0, then the thermal expansion coefficient of the shell must be less than that of the fiber ferrule, i.e., *α*_1_ < *α*_2_.

For a given FFPC sensing probe, *α*_1_, *α*_2_, and *L*_2_ have defined values. The proper static cavity length is referred to as *L*_10_. When the static length is *L*_10_, there is *dL*/*dT* = *α*_1_*L*_10_ − *α*_2_*L*_2_ = 0. In the case of *α*_1_ < *α*_2_, *dL*/*dT* will be smaller than zero when *L*_1_ < *L*_10_, and *dL*/*dT* will be greater than zero when *L*_1_ > *L*_10_. It is very interesting that the value of *dL*/*dT* could vary from a negative value to a positive value with *L*_1_ increasing and we can make *dL*/*dT* close to zero by adjusting the cavity length. So, we can obtain a temperature-insensitive FFPC sensing probe by two steps: (1) select the materials of the shell, core, and fiber ferrule according to α_1_ < α_2_, and (2) adjust *L* to let *dL*/*dT* be close to zero. We call this method structure compensation. It should be mentioned that the value of *dL*/*dT* can be obtained by experiments.

In experiments, to get a proper static cavity length for high thermal stability of the FFPC sensing probe, we first set an arbitrarily static cavity length. Then, we obtained interference spectroscopies at different temperatures and the value of Δ*L*/Δ*T*, where Δ*L* is the change of cavity length and Δ*T* is the change of temperature. If Δ*L*/Δ*T* < 0, we enlarged the cavity length *L*, and if Δ*L*/Δ*T* > 0, we reduced *L* until reaching the proper cavity length. To obtain the proper static cavity length, we needed to get the relative adjusting length to cavity length. The relative adjusting length is referred to as Δ*L*_R_. 

In order to get Δ*L*_R_, we only needed the value of Δ*L*/Δ*T* that can be obtained by experiments and the thermal expansion coefficient *α*_1_ of shell, and we did not need the exact values of *L*_1_, *α*_2_, and *L*_2_. The relative adjusting length Δ*L*_R_ can be determined by Equation (10):

Δ*L*_R_ = − (Δ*L*/Δ*T*)/*α*_1_ (10)

According to Equations (9) and (10), *L*_10_ = *L* + Δ*L*_R_, which is the proper static cavity length. Normally, we could not precisely adjust the length change to L_10_ at once, so two or three adjusting circles were needed.

From the above analysis, the key to applying structure compensation is how to get the change of cavity length ΔL. To get the value of ΔL by experiments, we acquired the interference spectroscopy at different temperatures by using a wideband source. The change of cavity length ΔL could be determined by the shift of interference spectroscopy Δλ by Equation (11) [31]:

ΔL/L = Δλ/λ_0_ (11)

where λ_0_ is the wavelength of a certain peak in the interference spectroscopy, and Δλ is the shift of interference spectroscopy in reference to λ_0_ with temperature change.

## 3. Composite Ferrule

Ceramic ferrule is commonly used in fiber sensors. Its thermal expansion coefficient is about 9.65 × 10^−6^ m/(m·°C). Most metals have a greater thermal expansion coefficient than that of ceramic ferrule. For example, the thermal expansion coefficient of nickel–copper alloy is about 14.4 × 10^−6^ m/(m·°C). So, it can be difficult to obtain properly pure materials with *α*_1_ < *α*_2_ for fabricating sensors.

To obtain *α*_1_ < *α*_2_, a feasible solution to obtain larger *α*_2_ is to use composite ceramic ferrule and a material with a large thermal expansion coefficient, i.e., epoxy, as shown in Figure 1b. The thermal expansion coefficient of the composite can be expressed as

(12) $$\alpha_{2}=k_{C}\alpha_{C}+k_{E}\alpha_{E}=\frac{L_{C}\alpha_{C}}{L_{C}+L_{E}}+\frac{L_{E}\alpha_{E}}{L_{C}+L_{E}}\;$$

where *k*_C_ and *k*_E_ are the proportion of the ceramic ferrule and epoxy, respectively; *α*_C_ and *α*_E_ are the thermal expansion coefficient of the ceramic ferrule and epoxy, respectively; *L*_C_ and *L*_E_ are the length of the ceramic ferrule and epoxy, respectively; and (*k*_C_ + *k*_E_) = 1. Due to *α*_E_ > *α*_1_, it is easy to get *α*_1_ < *α*_2_ by compositing.

**Figure 2:**
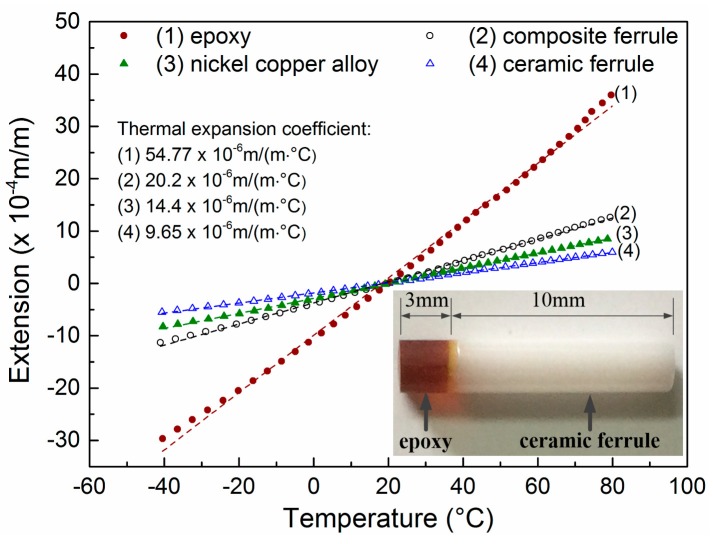
Experimental expansion coefficients of epoxy (**line 1**), composite ferrule (**line 2**), nickel–copper alloy (**line 3**), and ceramic ferrule (**line 4**). Inset is the photo of a composite ferrule sample.

The expansion coefficients of epoxy, nickel–copper alloy, ceramic ferrule, and composite ferrule were experimentally tested, as shown in Figure 2. *α*_C_ was about 9.65 × 10^−6^ m/(m·°C), and *α*_E_ was about 55 × 10^−6^ m/(m·°C). The dotted lines for each curve are linearly fit curves. The inset in Figure 2 is a composite ferrule sample of epoxy and ceramic ferrule. For the sample, *L_C_* = 10 mm, *L_E_* = 3 mm, and the calculated value of thermal expansion coefficient was about 20.06 × 10^−6^ m/(m·°C). While the experimental value was about 20.2 × 10^−6^ m/(m·°C), they agreed with each other well. This result indicates that the thermal expansion coefficient could be designed according to Equation (12).

Furthermore, it is worth noting that the curves in Figure 2 all have a good linearity in the temperature range of −20 to 60 °C, which satisfies most practical applications. Further, the thermal expansion coefficients of the tested materials can be considered as a constant, that is, (6) is reasonable. So, in our experiments, we tested all of the FFPC probes in the temperature range of −20 to 60 °C.

**Figure 3:**
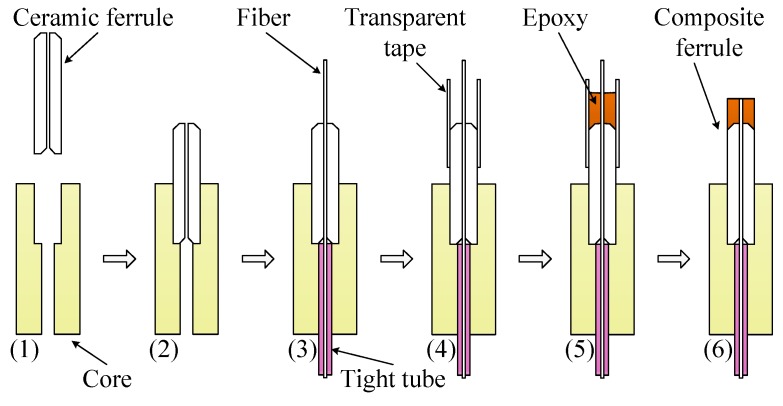
Process of fabricating a composite ferrule.

Figure 3 shows the process of fabricating a composite ferrule. First, a ceramic ferrule was pressed into a core, and then optical fiber was inserted into the ceramic ferrule (steps (1)–(3)). It should be noted that the part of the optical fiber above the end surface of the ceramic ferrule was more than 5 mm. Next, by using transparent tape, a fence was formed around it (step (4)), and then epoxy was injected into it (step (5)). In order to accelerate the curing of epoxy, it was put into a drying box at temperatures of 60–90 °C. After curing the epoxy, the transparent tape was removed, and the excess fiber was cut off. Then, the fiber with composite ferrule was grounded and polished (step (6)). It should be emphasized that the thermal expansion coefficient of the composite ferrule must be more than that of the shell. For ceramic ferrule with a length of 10 mm, the length of the epoxy part should not be less than 1.5 mm, at least.

## 4. Results and Discussion

To validate the structure compensation, we fabricated FFPC acoustic sensor probes. The sensing part of the probes was nickel foil with a thickness of 3 μm and a diameter of 9 mm. The materials of the shell and the core were nickel–copper alloy. The diameters of the shell and the core were 12.7 mm and 6 mm, respectively. A vent on the shell (as shown in Figure 1a) acted as pressure equalization. The vent, with a diameter of about 0.5 mm, was formed by laser drilling. The fiber ferrule was composite ferrule, as shown in the inset of Figure 2. In the probe, we controlled for *α*_1_ < *α*_2_.

**Figure 4:**
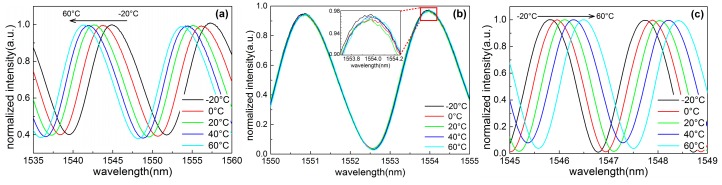
Normalized interference spectroscopy of a typical FFPC acoustic sensor probe with the static cavity length of about 96.5 μm (**a**), 386.95 μm (**b**), 621.5 μm (**c**) in a temperature range from −20 to 60 °C every 20 °C. The black arrows indicate the shift direction of the inference spectroscopy with the increasing temperature.

Figure 4 shows the normalized interference spectroscopies of a typical FFPC acoustic sensor probe with different static cavity lengths in a temperature range of −20 to 60 °C every 20 °C. At each testing temperature, the temperature was kept constant for more than 15 min. Then, the spectroscopy was acquired by a spectrometer.

From Figure 4a–c, the static cavity length of the FFPC probe is increasing from about 96.5 to 621.5 μm. From Figure 4a, we see an apparently blue shift of the interference spectroscopy, with temperature increasing for a short static cavity length of 96.5 μm, that is, the cavity length decreases with temperature increasing. The value of Δ*L*/Δ*T* is negative. When the static cavity length increases to 386.95 μm, we see that the interference spectroscopy almost overlaps each other during temperature change (see Figure 4b). The spectroscopy has a shift Δλ of ~15 pm for temperature change Δ*T* of 80 °C, namely, the spectroscopy has a shift rate of ~0.19 pm/°C, which is smaller than that of a single-mode microfiber Sagnac loop interferometer (~3 pm/°C) [32]. The change of cavity length ΔL is about 6 nm, and the value of Δ*L*/Δ*T* is about 0.075 nm/°C. When the static cavity length increases to 612.5 μm, we see a dramatic red shift of the interference spectroscopy with the temperature increasing. This indicates that the cavity length increases with the increasing temperature and the value of Δ*L*/Δ*T* is positive. These results agree well with the depiction of the thermal expansion model.

**Figure 5:**
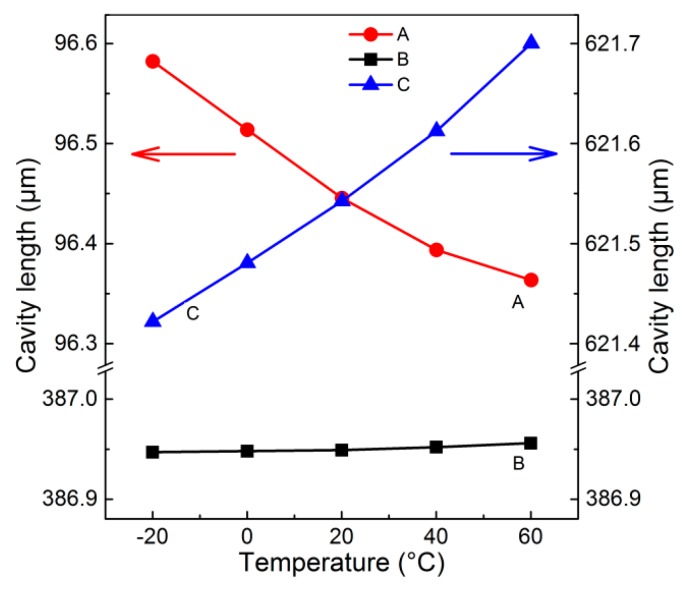
Cavity length-temperature curves of the typical FFPC acoustic sensor probe at different static cavity length of about 96.5 μm (**A**), 386.95 μm (**B**), and 621.5 μm (**C**).

According to interference spectroscopy shown in Figure 4, we can obtain the cavity length of the FFPC probe at every testing temperature point. Figure 5 shows the cavity length-temperature curves of the FFPC probe at different static cavity lengths. Curves A, B, and C correspond to Figure 4a–c, respectively. The scale of the cavity length axis in Figure 5 is proportional. The slope of the curves represents Δ*L*/Δ*T*. From Figure 5, we can intuitively observe that the value of Δ*L*/Δ*T* varies from a negative value to about zero until reaching a positive value with the static cavity length increasing.

After getting the value of Δ*L*/Δ*T*, it is easy to calculate the length that needs to be adjusted. For example, the average slope of curve C in Figure 4 is about 3.44 nm/°C. *α*_1_ of nickel–copper alloy is about 14.4 × 10^−6^ m/(m·°C). According to Equation (10), we get that Δ*L*_R_ is about −239 μm, and the minus symbol represents reducing the cavity length. So, the calculated possible proper cavity length *L*_New_ is about 382.5 μm, which is a predicted value. When the static cavity length is adjusted to the predicted value with several micrometers error, the probe shows high thermal stability (as shown in Figure 4b).

**Figure 6:**
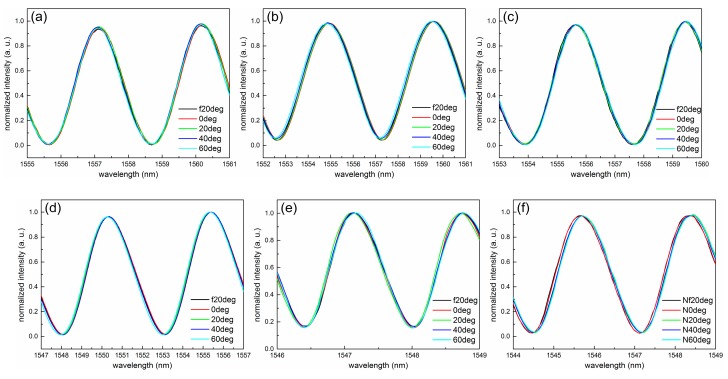
Normalized interference spectroscopy of six FFPC acoustic sensor probes with structure compensation at increasing temperatures from −20 to 60 °C every 20 °C.

By applying structure compensation, another six FFPC acoustic sensor probes were fabricated. Every probe was adjusted to its proper cavity length, which was 386.91 μm (Figure 6a), 258.89 μm (Figure 6b), 319.69 μm (Figure 6c), 239.54 μm (Figure 6d), 744.19 μm (Figure 6e), and 448.72 μm (Figure 6f). The interference spectroscopies of these probes with increasing temperature are shown in Figure 6. The curves in Figure 6 almost overlap each other. These results indicate that the structure compensation has good reproducibility.

**Figure 7:**
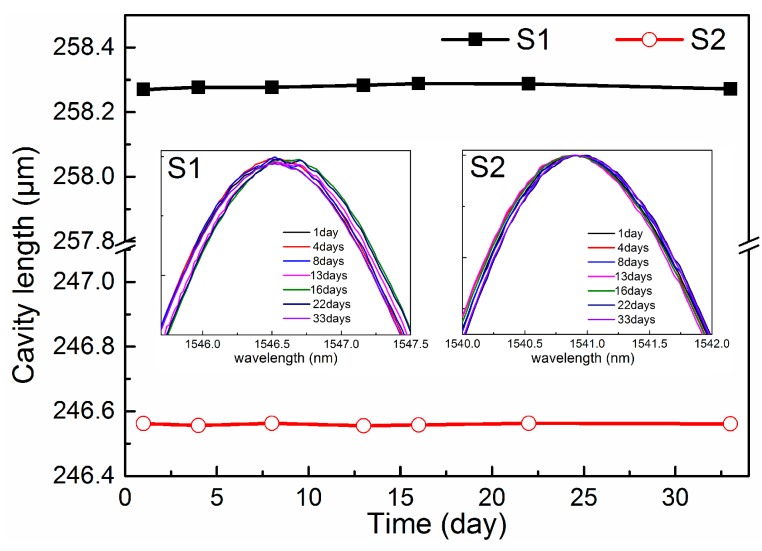
Cavity length-time curves of two typical samples. Insets are the interference spectroscopy of the two probes.

We also investigated the time-dependent stability of the FFPC acoustic sensor probe. Figure 7 shows the cavity length-time curve of two typical samples. The insets in Figure 7 are the interference spectroscopies of the two samples (S1 and S2). According to the interference spectroscopies, for sample S1, L = 258.252 μm, λ_0_ = 1546.541 nm, and maximum Δλ is 58 pm; so, the change of cavity length ΔL is about 9.7 nm. For sample S2, L = 245.772 μm, λ_0_ = 1540.913 nm, and maximum Δλ is 43 pm; so, the change of cavity length ΔL is about 6.9 nm. Thus, the cavity length has a change of about ±10 nm during about a month, which has limited impacts on the reliability of the sensors.

## 5. Acoustic Sensor

**Figure 8:**
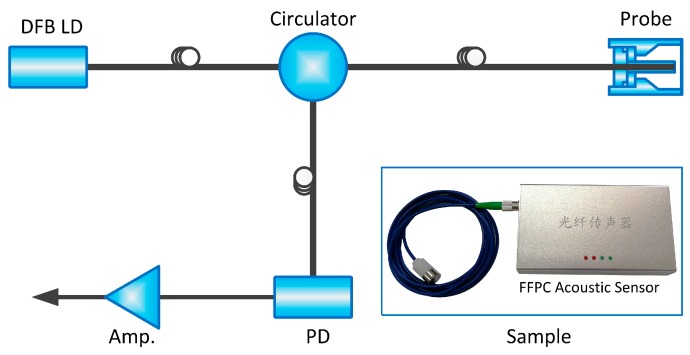
Schematic structure of the FFPC acoustic sensor. Inset in the lower right corner is the fabricated sample.

So far, it is worth emphasizing that the structure compensation provides a simple approach that allows the probe to be used in practical applications. If employing this probe to fabricate an acoustic sensor, the expensive tunable laser and complicated feedback system are no long required. To exhibit this advantage, an FFPC acoustic sensor was fabricated by only using a butterfly packaged DFB laser diode (LD), a circulator photo diode (PD), and an amplifier (Amp.). The schematic structure of the FFPC acoustic sensor is shown in Figure 8. The photo shown in the lower right corner in Figure 8 is the sample. It is obvious that the system is concise and tight. The principle of the acoustic sensor has been described in other papers [24,25,26].

**Figure 9:**
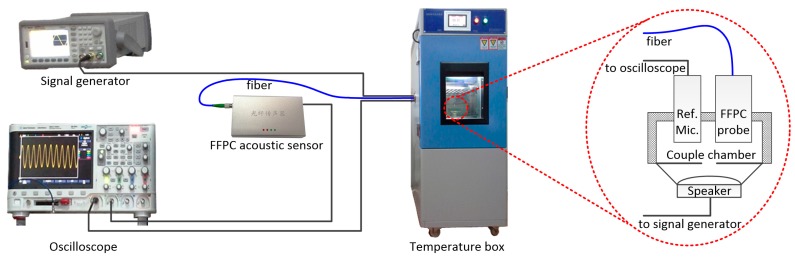
Experimental setup for investigating the effects of temperature on the FFPC acoustic sensor.

To investigate the effects of temperature on the FFPC acoustic sensor, it was transferred into a precisely controllable temperature box (model JT, made by Dongguan Jinte Instrument Co., Ltd., Dongguan, Guangdong, China). An electret condenser microphone (type 1/2 inch, made by Research Institute of Television and Electro-acoustics, Beijing, China) was employed as a reference. The output of the sensors was recorded by an oscilloscope (KEYSIGHT DSOX2024A). A sound wave was produced by a speaker driven by a signal generator (KEYSIGHT 33510B). The experimental setup is shown in Figure 9. The enlarged dot circle shows the details of the part of the testing system in the temperature box.

The output of the sensors under a 1-kHz sound wave excitation was acquired from −20 to 60 °C every 20 °C. The temperature was kept constant for more than 15 min at each testing temperature. Then, the data were recorded by oscilloscope.

**Figure 10:**
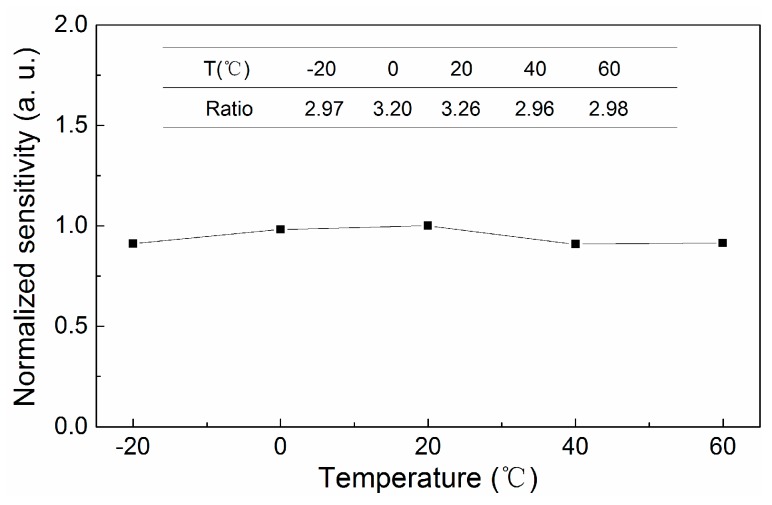
Normalized sensitivity-temperature curve of the FFPC acoustic sensor.

The ratio of the output of the FFPC acoustic sensor to that of the electret acoustic sensor has was calculated, as shown in the inset chart in Figure 10. The normalized sensitivity-temperature curve of the FFPC acoustic sensor was also obtained, as shown in Figure 10. The sensitivity had a relative change of 9.2% in the temperature range of −20 to 60 °C, which is acceptable in practical applications. The results show that the FFPC acoustic sensor with structure compensation has good thermal stability.

**Figure 11:**
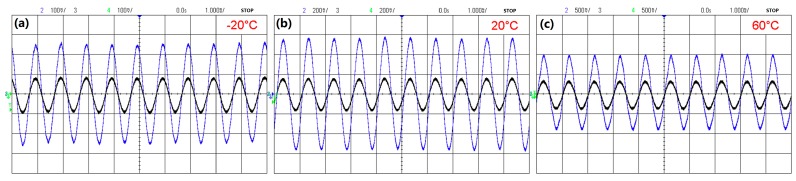
Outputs of the FFPC acoustic sensor and the reference condenser microphone at temperatures of −20 (**a**), 20, (**b**) and 60 °C (**c**). The larger signal (blue) is the output of the FFPC acoustic sensor, and the smaller signal (black) is the output of the condenser microphone.

Figure 11 shows the outputs of the FFPC acoustic sensor and the reference condenser microphone for a 1-kHz acoustic wave at different temperatures of −20, 20, and 60 °C. At each temperature, the FFPC acoustic sensor gave a sinusoidal wave with a period of 1 ms. The results indicate that the FFPC acoustic sensor works well with temperature change. Additionally, sensitivity of the FFPC acoustic sensor is about three times more than that of the condenser microphone.

**Figure 12:**
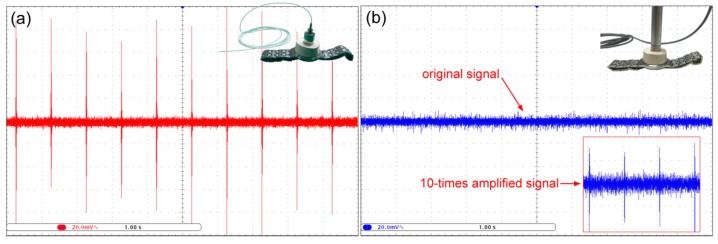
Output of an FFPC acoustic sensor (**a**) and a traditional condenser microphone (**b**) for hearing the sound of the second hand of a quartz watch.

Because of the high sensitivity of the FFPC acoustic sensor, it is suitable for detecting weak sound. To exhibit this ability, we put the sensing probe on the glass of a quartz watch and observed the output of the sensor by an oscilloscope. Figure 12a shows the sound signal of the working second hand. The signal is the original output of the FFPC acoustic sensor without any amplification. It shows clear pulses with a period of 1 s.

In contrast, a traditional condenser microphone (BK4190) can hardly detect the sound (see Figure 12b). When the output signal of the condenser microphone is amplified 10 times, pulse signals emerge (see inset with red box in Figure 10b). However, the signal to noise of the pulse signal is apparently smaller than that of the FFPC acoustic sensor. This result indicates that the optical sensor has better detection capabilities for weak acoustic signals than conventional condenser sensors.

The FFPC acoustic sensor also could detect low-frequency acoustic waves, while the optical microphone with feedback system [25] could not be detect quite low-frequency acoustic waves (less than 5 Hz) because the low-frequency signal would be judged as environmental changes and would be filtered.

**Figure 13:**
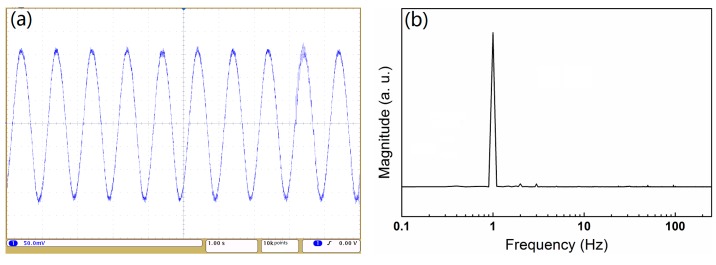
Time-domain signal of an FFPC acoustic sensor for a 1-Hz sound excitation (**a**) and FFT of a time-domain signal (**b**).

Figure 13a shows the time-domain signal of the FFPC acoustic sensor for a 1-Hz sinusoidal sound excitation with a sound pressure level of 94 dB. Figure 13b shows fast Fourier transformation (FFT) of the signal shown in Figure 13a. The FFT pattern shows a strong peak at 1 Hz. The result indicates that the FFPC acoustic sensor with structure compensation can detect infrasound waves.

## 6. Conclusions

In summary, a simple but effective method of structure compensation was proposed by the thermal expansion model to resolve the effects of temperature on the stability of an FFPC sensor for detecting dynamic signals. The results indicate that a temperature-insensitive FFPC sensing probe can be obtained by applying structure compensation in the temperature range of −20 to 60 °C. The change of the cavity length can be controlled with about 6 nm, with no pronounced effects on the sensors.

We applied the structure compensation to an FFPC acoustic sensor. Due to the inherent temperature insensitivity of the FFPC probe, the acoustic sensor had a simple architecture that did not use a tunable laser and feedback circuit. This method has promising practical applications in the medical, military, and industrial fields, such as communication in nuclear magnetic resonance, sound source locating, and structure condition monitoring.
